# Lipid Profile and Small Dense Low-Density Lipoprotein in Acute Coronary Syndrome Patients: Relationships to Demographic, Clinical, Angiographic, and Therapeutic Variables

**DOI:** 10.3390/jcm11226846

**Published:** 2022-11-20

**Authors:** Akshyaya Pradhan, Ravninder Kuka, Pravesh Vishwakarma, Wahid Ali, Marco Alfonso Perrone, Ferdinando Iellamo, Gaurav Chaudhary, Sharad Chandra, Rishi Sethi, Sudhanshu Dwivedi, Varun Narain, R. K. Saran

**Affiliations:** 1 Department of Cardiology, King George’s Medical University, Lucknow 226003, Uttar Pradesh, India; 2 Department of Cardiology, SPS Hospital, Ludhiana 141001, Punjab, India; 3 Department of Pathology, King George’s Medical University, Lucknow 226003, Uttar Pradesh, India; 4 Department of Cardiology and Cardio Lab, University of Rome Tor Vergata, 00133 Rome, Italy; 5 Department of Clinical & Preventive Cardiology, Medanta Hospital, Lucknow 226003, Uttar Pradesh, India

**Keywords:** acute coronary syndrome, atherosclerosis, small dense low-density lipoprotein, non-high-density lipoprotein cholesterol, sd-LDL

## Abstract

Background: Several lines of evidence have supported small dense low-density lipoproteins (sd-LDL) as a marker of cardiovascular disease. The present study assessed the relationship between lipid profile and sd-LDL levels with demographic, clinical, angiographic, and therapeutic variables in acute coronary syndrome (ACS) patients. Methods: This was a single-centre, prospective, cross-sectional study conducted from September 2014 to September 2015. Patients with a diagnosis of ACS were included in this study. High-density lipoprotein cholesterol (HDL-C) and low-density lipoprotein cholesterol (LDL-C) were determined by direct homogenous assay and sd-LDL levels were calculated using an earlier described equation by Srisawadi et al. Results: A total of 200 patients with a diagnosis of ACS were studied. Males constituted 78% of the population cohort and almost 45% of participants were aged <45 years. Patients aged ≤45 years displayed higher mean sd-LDL levels of 30.40 ± 14.18 mg/dL versus patients aged >45 years with mean sd-LDL levels of 28.01 ± 11.58 mg/dL, but the difference was not statistically significant (*p* = 0.19). Females also displayed higher mean sd-LDL levels, but the difference also failed to achieve statistical significance (30.95 ± 13.44 mg/dL and 28.54 ± 12.64, respectively; *p* = 0.185). Diabetics had higher mean sd-LDL levels (33.64 ± 13.01 mg/dL and 28.07 ± 12.60 mg/dL; *p* = 0.273) whilst smokers had lower mean levels (27.21 ± 12.12 mg/dL and 30.51 ± 13.21 mg/dL, respectively; *p* = 0.071). However, the ratio of sd-LDL/lb-LDL (large buoyant LDL) was significantly higher in diabetics (0.48 vs. 0.39; *p* = 0.023). In the angiography cohort (n = 88), single-vessel disease was the most predominant overall while among patients aged >45 years, triple-vessel disease was significantly higher (*p* = 0.005). Similarly, the sd-LDL levels were 33.12 ± 11.13 mg/dL, 27.68 ± 9.80 mg/dL, and 31.65 ± 15.26 mg/dL among patients with single, double, and triple-vessel disease and did not differ significantly (*p* = 0.262). Prior statin users had significantly lower mean sd-LDL levels of 24.79 ± 12.23 mg/dL compared to statin-naïve patients with a mean sd-LDL of 30.01 ± 12.79 mg/dL (*p* = 0.027). Non-HDL levels were also significantly lower in prior statin users (112.83 mg/dL vs. 128.9 mg/dL; *p* = 0.017). Conclusion: In this cohort of ACS patients, age, sex, diabetes, smoking, and the angiographic severity of coronary artery disease had no significant impact on sd-LDL levels, while prior statin usage led to significantly lower sd-LDL levels. Diabetic patients, however, did have significantly higher sd-LDL/lb-LDL ratios.

## 1. Introduction

Low-density lipoprotein cholesterol (LDL-C) is the primary cholesterol-carrying lipoprotein in human plasma. However, even when LDL-C levels are maintained within normal levels, the risk of cardiovascular events persists [1]. This observation has impelled the search for a more robust predictor of cardiovascular events. Small dense low-density lipoproteins (sd-LDL) have several features associated with atherogenesis: long residence time in plasma and greater oxidation potential, arterial proteoglycan binding, and permeability through the endothelial barrier—the early steps of atherogenesis. Atherogenicity potential increases with an increase in atherogenic lipoprotein particles and is lower if few particles are present. Thus, increased sd-LDL levels indicate a greater atherogenic risk, which may not be exposed by levels of LDL-C [2]. The landmark Framingham Offspring study similarly found sd-LDL as the most atherogenic lipoprotein among others such as total cholesterol (TC), triglycerides (TG), high-density lipoprotein cholesterol (HDL-C), direct LDL-C, LDL TG, remnant lipoprotein particle cholesterol, TG-rich lipoprotein cholesterol, and lipoprotein (a) [3]. Several other studies have documented the association between sd-LDL levels and cardiovascular risk [4,5,6,7].

The first homogenous assay for the detection of sd-LDL-C was described by Hirano et al. [8] in which heparin magnesium was used as a precipitating agent. The analytical procedure was later refined, allowing the separation of sd-LDL particles of sizes 15–20 nm and densities of 1.044–1.063 g/mL using standard equipment [9,10]. The method was again modified by Renjith et al. [11], which permitted the precipitation of lipoproteins of densities <1.044 g/mL using 40 U/mL heparin sodium salt and 30 mmol/L MnCl_2_. TC content in the supernatant (sd-LDL and HDL-C) was quantitated using a cholesterol assay kit, which permitted the calculation of sd-LDL as: sd-LDL = (TC in the supernatant -HDL-C). Srisawadi et al. [12] then developed an equation for the indirect calculation of sd-LDL. Here, sd-LDL-C (mg/dL) = 0.580 (non–HDL-C) + 0.407 (dLDL-C) − 0.719 (cLDL-C) − 12.05. Later, Samanta et al. [13] implemented the method described by Sriswadi et al. [12] and confirmed that calculated sd-LDL may be used as a substitute for estimated sd-LDL. The present study aimed to assess the relationship between lipid profile and sd-LDL with demographic, clinical, angiographic, and therapeutic variables in acute coronary syndrome (ACS) patients by indirectly calculating the sd-LDL levels using a previously formulated and validated equation.

## 2. Materials and Methods

### 2.1. Study Design and Patient Population

This was a single-centre, prospective, cross-sectional study conducted at a tertiary-care centre from September 2014 to September 2015. Patients aged 18 to 75 years who presented at the Emergency Department with de novo diagnosis of ACS were included in this study. The diagnosis of ACS was established on the basis of the third universal definition of myocardial infarction [14,15]. Patients with chronic renal failure, pre-existing liver pathology, familial dyslipidemia, triglyceride levels >400 mg/dL, moderate to severe valve stenosis, or adherence to oral contraceptive pills were excluded from the study. Chronic renal failure and liver pathology were defined as per standard definitions. Echocardiography was performed in all patients as per standard guidelines. Valve stenosis was defined according to the European Association of Echocardiography/American Society of Echocardiography guidelines [16]. Every patient was given the option of undergoing coronary angiography; however, only 88 patients underwent the procedure. The study protocol was approved by institutional ethics committee and conducted in compliance with the ethical standards of the Institutional Ethics committee as with the Helsinki Declaration. The study patients were not offered any other lipid lowering medication other than the statin therapy prescribed as per the study protocol. All patients provided written informed consent for study participation prior to the commencement of the study.

### 2.2. Study Procedure

A fasting blood sample was obtained from each patient as soon as feasible, although no later than 24 h after hospitalization. The lipid tests included TC, HDL-C, TG, LDL-C, and its small subfraction, sd-LDL. TC and TG were estimated using readymade kits (Accurex Biomedical Pvt. Ltd., Mumbai, Maharasthra, India) based on the enzymatic method (CHOD-POD and GPO-POD, respectively). HDL-C and LDL-C were determined by direct homogenous assay (Accurex Biomedical Pvt. Ltd., Mumbai, India). Calculated low-density lipoprotein cholesterol (cLDL-C) was determined using the Friedewald formula [17]:

LDL-C (mg/dL) = TC − (HDL-C) − (TG/5)

where TG divided by 5 represents the very-low-density lipoprotein (VDLC) cholesterol concentration. The difference between LDL-C and direct low-density lipoprotein cholesterol (dLDL-C) has been ascribed to the presence of sd-LDL. Srisawadi’s method of estimation of sd-LDL using the Friedewald equation and stepwise regression equation is as follows [17]:

sd-LDL (mg/dL) = 0.580 (non-HDL-C) + 0.407 (dLDL-C) − 0.719 (cLDL-C) − 12.05

### 2.3. Study Definitions

ACS was defined according to the third universal definition of myocardial infarction [14]. Acute myocardial infarction was diagnosed by detection of the rise and/or fall of cardiac biomarker values with at least one value above 99th percentile of the upper reference limit with at least one of the following: (i) symptoms of ischemia; (ii) new or presumed-new significant ST-segment, T-wave changes, or new left bundle branch block (LBBB); (iii) development of pathological Q waves on electrocardiography; (iv) imaging evidence of a new loss of viable myocardium or a new regional wall motion abnormality; or (v) identification of an intracoronary thrombus by angiography. Coronary artery disease (CAD) was defined as stenosis of one or more coronary artery branches with ≥50% diameter luminal narrowing as observed on coronary angiography. Unstable angina was usually considered when cardiac biomarkers were undetectable in the bloodstream hours after the initial onset of ischemic pain. It presents with one or more of the following characteristics: (i) rest angina (usually lasting >20 min); (ii) new-onset (less than 2 months prior) severe angina; and (iii) a crescendo pattern of occurrence (increasing in intensity, duration, frequency, or any combination of these factors).

### 2.4. Data Collection

Data on demographics, laboratory investigations, clinical, angiographic, and therapeutic variables were collected for all patients from the patients’ medical records at the initial assessment.

### 2.5. Statistical Analysis

Continuous data are presented as mean ± standard deviation. Categorical data are presented as counts and percentages. Student’s *t*-test was used to compare means between two groups, whereas ANOVA was used to examine differences in the descriptive characteristics of the study population. All statistical analysis was done using Statistical Package for Social Sciences (SPSS; Chicago, IL, USA) program, version 20.

## 3. Results

### 3.1. Demographics of Study Patients

A total of 234 patients were screened, but 34 did not meet the inclusion criteria. Thus, only 200 patients were included in this study. Males predominantly comprised the study population. Females were three times less prevalent than males. Risk factors such as diabetes and smoking were prevalent among 36 (18.0%) and 87 (43.5%) patients, respectively. Severity of CAD was assessed in 88 of the 200 patients, as only 88 patients underwent coronary angiography. Single-vessel disease (48.9%) was more common than double-vessel disease (22.7%) and triple-vessel disease (28.4%). The demographics of the study patients are outlined in Table 1. ACS was more prevalent in males ≤45 years compared to females ≤45 years. Single-vessel disease was more common (64.7%) in patients aged ≤45 years compared to patients aged >45 years (38.9%); however, triple-vessel disease was significantly more common (40.7%) in patients ≤45 years compared to patients aged >45 years (8.8%). The demographics of the study patients according to age groups ≤45 years and >45 years are detailed in Table 2.

### 3.2. Lipid Profile According to Patient Age and Gender

**Figure 1:**
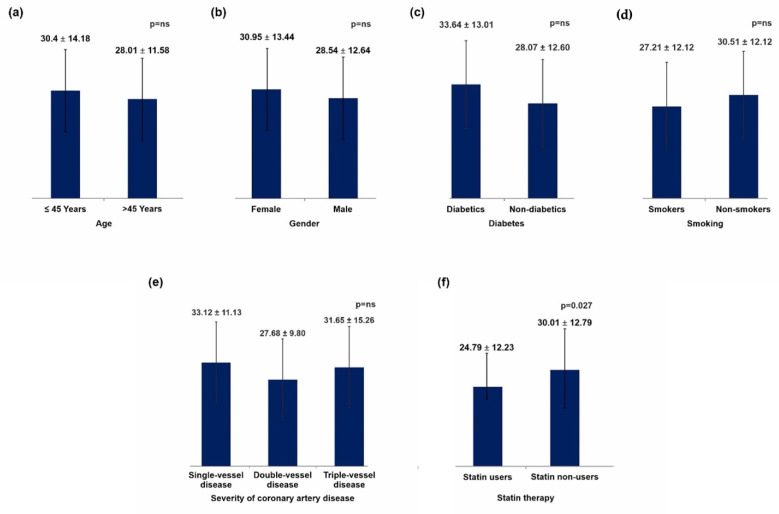
Sd-LDL levels according to demographic, clinical, angiographic, and therapeutic variables. (**a**). sd-LDL levels according to Age. (**b**). sd-LDL levels according to gender. (**c**). sd-LDL levels according to diabetes status. (**d**). sd-LDL levels according to smoking status. (**e**). sd-LDL levels according to severity of coronary artery disease. (**f**). sd-LDL levels according to baseline statin usage.

Sd-LDL levels were 30.40 ± 14.18 mg/dL and 28.01 ± 11.58 mg/dL for patients aged ≤45 years and >45 years, respectively, and 30.95 ± 13.44 mg/dL and 28.54 ± 12.64 mg/dL for females and males, respectively. However, these findings were not statistically significant. Relations between sd-LDL, age, and gender are displayed in Figure 1a,b, respectively. Lipid profiles according to patient age and gender are demonstrated in Table 3.

### 3.3. Lipid Profile Comparison between Diabetics versus Non-Diabetics and Smokers versus Non-Smokers

Sd-LDL levels were 33.64 ± 13.01 mg/dL and 28.07 ± 12.60 mg/dL for diabetics and non-diabetics, as shown in Figure 1c, and 27.21 ± 12.12 mg/dL and 30.51 ± 13.21 mg/dL for smokers and non-smokers, as displayed in Figure 1d. Similarly, the sd-LDL levels were 33.12 ± 11.13 mg/dL, 27.68 ± 9.80 mg/dL, and 31.65 ± 15.26 mg/dL for single-vessel disease, double-vessel disease, and triple-vessel disease, respectively, as shown in Figure 1e. The lipid profile comparison between diabetics versus non-diabetics and smokers versus non-smokers is detailed in Table 4. Lipid profiles according to angiographic findings are shown in Table 5.

### 3.4. Lipid Profile Comparison According to Patient’s Statin Therapy

Sd-LDL levels for statin users and statin non-users were 24.79 ± 12.23 mg/dL and 30.01 ± 12.79 mg/dL, respectively, as illustrated in Figure 1f (*p* = 0.027).TC levels were 149.32 ± 36.66 mg/dL and 170.61 ± 41.24 mg/dL (*p* = 0.005), and dLDL-C levels were 90.43 ± 26.77 mg/dL and 103.76 ± 32.17 mg/dL (*p* = 0.022) for statin users and statin non-users, respectively.Non-HDL levels were similarly significantly lower with prior statin users (*p* = 0.017). A lipid profile comparison according to patients’ statin therapy is delineated in Table 6.

## 4. Discussion

Several studies have explored the association between sd-LDL levels and cardiovascular risk [4,5,6,7]. The current study attempted to further explore the relationship between lipid profiles and sd-LDL levels with age, gender, clinical, and therapeutic variables. The study findings revealed lower mean sd-LDL levels in statin users as compared to statin non-users. This finding was statistically significant. Statin users also had lower levels of non-HDL-c. It is important to note that non-HDL-c is a marker of total atherogenic burden and is now a secondary target after LDL in many contemporary guidelines. The study also revealed higher mean sd-LDL levels in: patients ≤45 years compared to patients >45 years; females compared to males; diabetics compared to non-diabetics; non-smokers compared to smokers; and patients with single-vessel disease compared to patients with double- or triple-vessel disease. These findings were not statistically significant. However, sd-LDL ratios were significantly higher in diabetic patients, indirectly indicating a higher sd-LDL (atherogenic) burden in vasculature.

In recent times, India has witnessed earlier onset of ACS in younger patients less than 45 years old. Indians develop ACS 5–10 years earlier than the Western population with 5–10-fold higher occurrence in patients younger than 40 years. Moreover, of the total incidence of ACS, 25% occurs in patients younger than 40 years and 50% in patients younger than 50 years [18]. In view of these statistics, the cut-off age of 45 years was determined for the current study. The early onset of ACS in the Indian population validates the assessment of the relationship between age and mean sd-LDL levels in ACS patients. Gender was another variable whose association with mean sd-LDL levels was assessed. In the current study, we observed three times less prevalence of females compared to males. Females comprised 22% of the study population whereas males predominantly comprised 78%. This finding is line with the study by Tsai et al. [19] whose study population included 92.9% males and 7.1% females. Similarly, Reda et al. [20] documented a study population comprised of 81% males and 19% females. A second finding revealed by the current study was the higher prevalence of ACS among males compared to females in patients both ≤45 years and >45 years. Moreover, an earlier study [21] revealed mean sd-LDL levels of 11.6 ± 5.9 mg/dL in females compared to 14.8 ± 7.2 mg/dL in males. Females are expected to have lower sd-LDL levels due to lower levels of hepatic lipase activity in females [22]. However, the current study revealed contradictory observations.

Hepatic lipase is commonly increased in subjects with type 2 diabetes and consequently, the prevailing metabolic conditions favour the formation of sd-LDL particles [23]. A Japanese study [24] investigated the prevalence of sd-LDL and abnormal glucose regulation in ACS patients. The study findings revealed prevalence of sd-LDL in 60% of the diabetics and 50% of the non-diabetics. Moreover, sd-LDL levels were prevalent in 61% of patients with glucose intolerance and 42% of patients with normal glucose tolerance. The authors concluded a close association between abnormal glycometabolism and highly atherogenic sd-LDL particles. Similarly, a Chinese study [25] associated elevated sd-LDL levels with greater cardiovascular risk in diabetic patients compared to non-diabetic patients. In line with these findings, the current study revealed higher mean sd-LDL levels in diabetics compared to non-diabetics. Additionally, the current study revealed that sd-LDL ratios were significantly different between diabetics and non-diabetics. Such a difference was not observed among other lipid parameters.

The pathology of CAD is multifaceted with a wide spectrum of modifiable and non-modifiable risk factors. Recent studies have revealed LDL-C as a risk factor. However, patients with normal LDL levels are still at risk of CAD. This observation has led to the hypothesis that sd-LDL might correlate more strongly with the severity of CAD. An Indian study [21] indicated higher mean sd-LDL levels in patients with coronary stenosis compared to patients without coronary stenosis (16.3 ± 6.8 versus 10.1 ± 5.7 mg/dL, respectively). Moreover, there was significant correlation between mean sd-LDL and severity of CAD as assessed by the SYNTAX score, with mean sd-LDL levels in low, intermediate, and high SYNTAX scores as 15.0 ± 5.8, 20.1 ± 6.7, and 22.7 ± 7.3 mg/dL, respectively. In line with these findings, the CUREUS-8 documented mean sd-LDL levels of 7.2 ± 6.8 mg/dL in patients without CAD compared to 16.7 ± 11.1 mg/dL in patients with CAD [26]. Another study [25] indicated that elevated levels of plasma sd-LDL were associated with an increased risk of major adverse cardiovascular events among diabetic patients with proven CAD.

Non-HDL-cholesterol comprises cholesterol carried by all potentially atherogenic particles such as intermediate-density lipoprotein (IDL), VLDL and its remnants, chylomicron particles, chylomicron remnants, and Lp(a), hence its use as a cardiovascular biomarker. It is advantageous in comparison to LDL in that it can be measured in either the fasting or non-fasting state [27]. Furthermore, non-HDL cholesterol can be directly calculated from values in routine lipid panels at no additional expense [28]. Findings from our study revealed that statin therapy significantly improved non-HDL as well as LDL levels. The Multinational Cardiovascular Risk Consortium revealed that non-HDL cholesterol concentrations in blood are strongly associated with long-term risk of atherosclerotic cardiovascular disease [29].

### Study Limitations

There were a few limitations of the study that deserve mentioning. Firstly, the sample size was small. Secondly, adherence to statin therapy by some patients could have acted as a confounding factor and led to variable results. However, we attempted to eliminate this error by segregating statin users and statin non-users. However, this led to a reduction in the study sample. Thirdly, the study lacked a control population. Fourthly, case–control studies with larger sample sizes in each group are needed for further verification of the role of sd-LDL as an independent risk factor for predicting CAD in young and elderly ACS patients. Fifthly, ox-LDL or s-LOX1 are important biomarkers [30]. The measurement and role that these biomarkers play in the process of atherosclerosis could have been assessed in this study. Lastly, the literature evidences an association between body mass index and sd-LDL [31]. However, body mass index was not assessed in the current study.

## 5. Conclusions

In this predominantly male cohort of ACS patients, age, sex, diabetes, smoking, and angiographic severity of coronary artery disease had no impact on sd-LDL levels, though they trended towards higher levels in younger patients, females, and diabetics. Only prior statin users had significantly lower sd-LDL levels. However, diabetic patients had significantly higher sd-LDL/lb-LDL ratios.

## Figures and Tables

**Table 1:** Demographics of study patients.

Variable	Patients (n = 200)
Males	156 (78.0%)
Females	44 (22.0%)
Diabetic	36 (18.0%)
Non-diabetic	164 (82.0%)
Smoker	87 (43.5%)
Non-smoker	113 (56.5%)
Single-vessel disease *	43/88 (48.9%)
Double-vessel disease *	20/88 (22.7%)
Triple-vessel disease *	25/88 (28.4%)
Anterior wall myocardial infarction	78 (39.0%)
Inferior wall myocardial infarction	91 (45.5%)
Non-ST-segment myocardial infarction	31 (15.5%)

All data are expressed as number (percentage). * Severity of CAD was assessed in 88 of the 200 patients.

**Table 2:** Demographics according to age.

Variable	≤45 Years (n = 89)	>45 Years (n = 111)	*p* Value
Males	75 (84.3%)	81 (73.0%)	0.055
Females	14 (15.7%)	30 (27.0%)	
Diabetic	13 (14.6%)	23 (20.7%)	0.263
Non-diabetic	76 (85.5%)	88 (79.3%)	
Smoker	36 (40.4%)	51 (46.0%)	0.436
Non-smoker	53 (59.6%)	60 (54.1%)	
Single-vessel disease	22/34 (64.7%)	21/54 (38.9%)	0.005
Double-vessel disease	9/34 (26.5%)	11/54 (20.4%)	
Triple-vessel disease	3/34 (8.8%)	22/54 (40.7%)	
Anterior wall myocardial infarction	32 (36.0%)	46 (41.4%)	0.715
Inferior wall myocardial infarction	42 (47.2%)	49 (44.1%)	
Non-ST-segment myocardial infarction	15 (16.9%)	16 (14.4%)	

All data are expressed as number (percentage). *p* value < 0.05 was considered statistically significant. Severity of CAD was assessed in 88 of the 200 patients.

**Table 3:** Lipid profile according to patient age and gender.

Variable	≤45 Years (n = 89)	>45 Years (n = 111)	*p* Value	Females (n = 44)	Males (n = 156)	*p* Value
TC, mg/dL	170.58 ± 46.28	163.73 ± 36.53	0.244	169.69 ± 38.00	165.96 ± 42.13	0.597
HDL, mg/dL	40.44 ± 10.57	39.98 ± 8.47	0.728	41.75 ± 10.78	39.74 ± 9.02	0.214
cLDLc, mg/dL	104.58 ± 36.76	98.88 ± 30.66	0.328	100.54 ± 30.86	102.39 ± 34.32	0.750
TG, mg/dL	118.22 ± 49.96	114.84 ± 51.95	0.643	126.59 ± 65.07	113.46 ± 46.07	0.131
Non-HDL, mg/dL	130.13 ± 40.81	122.70 ± 33.03	0.157	127.02 ± 33.99	125.72 ± 37.64	0.836
dLDLc, mg/dL	103.79 ± 34.96	99.42 ± 28.69	0.333	102.29 ± 27.96	101.10 ± 32.66	0.826
sd-LDL-C, mg/dL	30.40 ± 14.18	28.01 ± 11.58	0.190	30.95 ± 13.44	28.54 ± 12.64	0.273
lbLDL-C, mg/dL	73.43 ± 25.41	71.46 ± 20.33	0.542	71.45 ± 20.98	72.59 ± 23.21	0.769
sd-LDL-C: lbLDL-C ratio	0.42 ± 0.11	0.40 ± 0.18	0.433	0.45 ± 0.23	0.40 ± 0.18	0.089

TC—total cholesterol, HDL—high-density lipoprotein, cLDLC—calculated low-density lipoprotein cholesterol, TG—triglycerides, dLDLc—direct low-density lipoprotein cholesterol, sd-LDL-C—small dense low-density lipoprotein cholesterol, lbLDLc—large buoyant low-density lipoprotein cholesterol. All data are expressed as mean ± standard deviation. *p* value <0.05 was considered statistically significant.

**Table 4:** Lipid profile comparison between diabetics versus non-diabetics and smokers versus non-smokers.

Variable	Diabetics (n = 164)	Non-Diabetics (n = 36)	*p* Value	Smoker (n = 80)	Non-Smoker (n = 106)	*p* Value
TC, mg/dL	168.19 ± 35.63	166.47 ± 42.41	0.597	161.82 ± 41.37	170.59 ± 40.82	0.136
HDL, mg/dL	37.98 ± 10.47	40.67 ± 9.17	0.214	40.44 ± 8.83	39.99 ± 9.93	0.739
cLDL-C, mg/dL	102.68 ± 27.37	101.84 ± 34.78	0.894	100.00 ± 33.95	103.49 ± 33.28	0.469
TG, mg/dL	136.49 ± 59.43	111.92 ± 48.00	0.131	109.59 ± 48.50	121.55 ± 52.41	0.100
Non-HDL, mg/dL	131.31 ± 30.30	124.84 ± 38.04	0.836	121.84 ± 37.39	129.22 ± 36.15	0.160
dLDL-C, mg/dL	106.58 ± 27.78	100.22 ± 32.37	0.826	98.78 ± 31.20	103.35 ± 31.94	0.312
sd-LDL-C, mg/dL	33.64 ± 13.01	28.07 ± 12.60	0.273	27.21 ± 12.12	30.51 ± 13.21	0.071
lbLDL-C, mg/dL	72.93 ± 1.100	72.21 ± 23.20	0.769	71.52 ± 22.14	72.97 ± 23.19	0.655
sd-LDL-C: lbLDL-C ratio	0.48 ± 0.21	0.39 ± 0.19	0.023	0.38 ± 0.18	0.43 ± 0.21	0.103

TC—total cholesterol, HDL—high-density lipoprotein, cLDLC—calculated low-density lipoprotein cholesterol, TG—triglycerides, dLDLc—direct low-density lipoprotein cholesterol, sd-LDL-C—small dense low-density lipoprotein cholesterol, lbLDLc—large buoyant low-density lipoprotein cholesterol. All data are expressed as mean ± standard deviation. *p* value < 0.05 was considered statistically significant.

**Table 5:** Lipid profile according to angiographic findings *.

Variable	SVD (n = 37)	DVD (n = 17)	TVD (n = 25)	*p* Value
TC, mg/dL	177.82 ± 31.17	169.65 ± 33.19	164.61 ± 50.03	0.364
HDL, mg/dL	40.00 ± 10.10	42.77 ± 9.06	39.24 ± 8.06	0.422
cLDL-C, mg/dL	109.05 ± 28.35	104.05 ± 24.03	97.02 ± 40.19	0.322
TG, mg/dL	126.65 ± 55.41	121.21 ± 46.81	132.04 ± 55.22	0.796
Non-HDL, mg/dL	135.00 ± 29.69	126.90 ± 29.18	125.46 ± 45.23	0.484
dLDL-C, mg/dL	110.03 ± 26.96	98.16 ± 22.32	100.08 ± 36.69	0.222
sd-LDL-C, mg/dL	33.12 ± 11.13	27.68 ± 9.80	31.65 ± 15.26	0.262
lbLDL-C, mg/dL	76.81 ± 20.23	70.48 ± 14.89	68.41 ± 25.74	0.239
sd-LDL-C: lbLDL-C ratio	0.45 ± 0.21	0.38 ± 0.14	0.47 ± 0.21	0.241

* Only 88 patients who underwent coronary angiography. SVD—single-vessel disease, DVD—double-vessel disease, TVD—triple-vessel disease, TC—total cholesterol, HDL—high-density lipoprotein, cLDLC—calculated low-density lipoprotein cholesterol, TG—triglycerides, dLDLc—direct low-density lipoprotein cholesterol, sd-LDL-C—small dense low-density lipoprotein cholesterol, lbLDLc—large buoyant low-density lipoprotein cholesterol. All data are expressed as mean ± standard deviation. *p* value < 0.05 was considered statistically significant.

**Table 6:** Lipid profile comparison according to patients’ statin therapy.

Variable	Statin Users (n = 36)	Statin Non-Users (n = 164)	*p* Value
TC, mg/dL	149.32 ± 36.66	170.61 ± 41.24	0.005
HDL, mg/dL	38.71 ± 10.30	40.51 ± 9.25	0.301
cLDL-C, mg/dL	90.92 ± 25.47	104.45 ± 34.66	0.028
TG, mg/dL, mg/dL	104.25 ± 40.27	119.00 ± 52.77	0.116
Non-HDL, mg/dL	112.83 ± 31.09	128.90 ± 37.39	0.017
dLDL-C, mg/dL	90.43 ± 26.77	103.76 ± 32.17	0.022
sd-LDL-C, mg/dL	24.79 ± 12.23	30.01 ± 12.79	0.027
lbLDL-C, mg/dL	65.63 ± 18.03	73.81 ± 23.38	0.778
Sd-LDL-C: lbLDL-C ratio	0.37 ± 0.19	0.41 ± 0.20	0.970

TC—total cholesterol, HDL—high-density lipoprotein, cLDL-C—calculated low-density lipoprotein cholesterol, TG—triglycerides, dLDLc—direct low-density lipoprotein cholesterol, sd-LDL-C—small dense low-density lipoprotein cholesterol, lbLDL-C—large buoyant low-density lipoprotein cholesterol. All data are expressed as mean ± standard deviation. *p* value < 0.05 was considered statistically significant.

## Data Availability

The data presented in this study are available on request from the corresponding author.
